# Factors Influencing Removal of Trichloroethylene in a Zero-Valent Iron Fenton System

**DOI:** 10.3390/nano15070558

**Published:** 2025-04-05

**Authors:** Yangyang Sun, Shichao Liang, Pengfei Li

**Affiliations:** 1 College of Geographical Science, Harbin Normal University, Harbin 150000, China; 2 Cooperative Economic Guidance Department, Suihua Agriculture and Rural Bureau, Suihua 152000, China

**Keywords:** trichloroethylene (TCE), zero-valent iron (ZVI), Fenton process, groundwater

## Abstract

Trichloroethylene (TCE), a volatile organic compound commonly used as a solvent, is frequently detected in contaminated groundwater. In the zero-valent iron (ZVI) Fenton process, TCE can be eventually dechlorinated into non-toxic products, which is mainly caused by hydroxyl radicals derived from H_2_O_2_. However, some key factors in the dechlorination of TCE in the zero-valent iron Fenton process have not been studied clearly. In the present study, the effects of the initial TCE concentration, initial H_2_O_2_ concentration, dosage of ZVI, initial pH, and temperature on TCE degradation in the ZVI Fenton process were studied. In addition, the structure and surface morphology of the ZVI used in this study were analyzed through scanning electron microscopy (SEM), N_2_ adsorption–desorption, and X-ray diffractometry (XRD). The experimental results demonstrated that the dosage of ZVI and initial H_2_O_2_ concentration had obvious impacts on TCE degradation. At a ZVI dosage of 2 g/L and an initial H_2_O_2_ concentration of 0.53 mol/L, more than 97% of TCE could be degraded within 24 h at 25 °C. We found that the ZVI Fenton process could efficiently degrade TCE at a broad pH range and room temperature, making it applicable to groundwater remediation. TCE degradation was associated with Fe^2+^ concentration. Spectroscopic analyses indicated that the oxide film formed on the ZVI surface was associated with Fe^2+^ concentration in enhanced TCE dechlorination. The ZVI Fenton process could work at a wide range of TCE concentrations (0–200 mg/L).

## 1. Introduction

Trichloroethylene (TCE), a colorless liquid with volatile properties, is extensively used as an organic solvent and is widely used in printing and dyeing processes, the cleaning industry, degreasing processes, and other chemical fields [1]. TCE is one of 129 priority pollutants and has attracted significant attention due to its characteristics of high toxicity, volatility, and long-term residual persistence [2]. Chlorinated organic pollutants have been detected in groundwater and soil in many countries and cities, posing a global ecological problem. These pollutants can persist in the environment for long periods of time and accumulate in organisms through the food chain, leading to significant risks to human health and the environment [3]. Previous studies have verified that humans could suffer from symptoms such as liver damage, paralysis, and cancer under the stress of high TCE levels [4,5]. In numerous species and strains of experimental animals, TCE has been demonstrated to be carcinogenic at multiple sites [4]. Moreover, TCE has been reported to influence the microbial community diversity and nitrogen cycle in Mollisol, which is a deep, organic-rich, and highly fertile soil type known for its black topsoil and excellent agricultural productivity [6,7]. The soil quality deteriorated and the organic matter decomposition and mineral nutrient cycling were impeded in Mollisol when the concentration of TCE exceeded 10 mg/kg, caused by its impact on the soil’s microbial biomass [6]. Therefore, investigating efficient TCE degradation pathways holds great practical significance. Many plans have been proposed to treat and restore chlorine-containing contaminated underground water, including gas stripping, physical adsorption, chemical oxidation or reduction [8,9,10], and hydrodechlorination [11]. Among various chemical oxidation techniques, the Fenton method has garnered significant interest due to its potent oxidizing capabilities [12]. In the classic Fenton method, hydrogen peroxide is catalyzed by ferrous iron to produce hydroxyl radicals that react with pollutants [13].

However, the pH range for the Fenton process is restricted to acidic conditions. Fe(II) is oxidized to Fe(III) in the presence of hydrogen peroxide, which acts as the oxidant species. Under alkaline conditions, Fe(III) ions hydrolyze and form ferric hydroxide precipitates. This precipitation removes Fe(III) from the solution, preventing further catalytic reactions with hydrogen peroxide, thereby terminating the Fenton reaction prematurely [14]. Moreover, hydroxyl radicals from the Fenton process, as powerful and non-selective oxidants that can even corrode cement [15], are destructive to the environmental medium itself. Therefore, a number of studies, including Fenton-like reactions and modified Fenton reactions, have been carried out to improve the limitations of the classic Fenton process.

Fenton processes using catechol [16] and thioglycolic acid [17] have been reported to degrade organic complexes at a neutral pH by accelerating the Fe(III)/Fe(II) redox cycle. Heterogeneous Fenton processes using solid iron sources such as goethite [18], magnetite [19], hematite [20], etc., have been a new research direction. Heterogeneous Fenton processes based on schwertmannite can enhance sludge’s dewaterability over a wide pH range [21].

Recently, ZVI has gained widespread attention for groundwater remediation due to its high reactivity and large surface area [22]. Pure ZVI has difficulty completely dechlorinating chlorine-containing contaminants, so it is usually combined with other chemical agents in contaminant degradation. Hydrodechlorination reactions with ZVI provide a fresh perspective for the degradation of chlorine-containing contaminants [23,24,25]. Wang et al. demonstrated that the heterogeneous Fenton process using goethite is highly pH-dependent, with optimal performance at pH 3, as higher pH levels lead to Fe precipitation and reduced catalytic efficiency [23]. In addition, Tiar et al. further confirmed that both homogeneous and heterogeneous photo-Fenton degradation of 4-nitrophenol are most effective under acidic conditions, with the degradation ratios declining significantly as the pH increases due to Fe(OH)₃ formation [24]. Moreover, heterogeneous Fenton processes using zero-valent iron (ZVI) instead of aqueous Fe(II) can reduce the disadvantages of metal loss in the classic Fenton process. ZVI acts as a slow-release source of aqueous Fe(II) [26]. The ZVI Fenton process has been reported to be used to treat organic wastewater produced by pharmaceutical factories. Using the ZVI Fenton process, the removal efficiency of sulfamethazine at pH 3 greatly exceeded that at other pH values [27,28]. However, the difference in initial pH scarcely influences the dechlorination ratio of molinate [29]. Thus, the influence of initial pH on the dechlorination ratio of TCE need to be further investigated.

In addition, some other key factors in dechlorination of TCE in the zero-valent iron Fenton process have not been investigated clearly; those that have been investigated were demonstrated to have effects on TCE dechlorination in the Cu(II) Fenton process [30] and sulfide-modified nZVI process [26]. Thus, studying the efficient TCE degradation in the ZVI Fenton process under different conditions is of a great importance.

This study aims to systematically investigate the key factors influencing the degradation and dechlorination efficiency of trichloroethylene (TCE) in the ZVI/H_2_O_2_ system, including initial TCE concentration, initial H_2_O_2_ concentration, ZVI dosage, initial pH, and temperature. Additionally, it explores the underlying mechanisms of TCE degradation in the ZVI-Fenton process. To gain deeper insights into the reaction process, advanced characterization techniques such as scanning electron microscopy (SEM), N_2_ adsorption–desorption analysis, and X-ray diffractometry (XRD) were employed to compare the morphology and structural changes in ZVI before and after the reaction. By monitoring TCE degradation ratios, dechlorination efficiency, and Fe(II) variations throughout the experimental process, this study seeks to identify optimal conditions for maximizing TCE removal efficiency. The findings not only contribute to a better understanding of the ZVI-Fenton system but also provide a scientific basis for developing more effective and sustainable remediation strategies for TCE-contaminated environments.

## 2. Experimental

### 2.1. Chemicals and Reagents

All chemicals and reagents used in this study were of analytical grade or higher. ZVI was purchased from Höganäs Environmental Solutions, LLC. Trichloroethylene (TCE, C_2_HCl_3_ ≥ 99.0%) purchased from Benchmark Chemical Reagent Ltd. Co., Tianjin, China. Hydrogen peroxide was purchased from Beilian Fine Chemicals Ltd. Co., Tianjin, China. Sodium hydroxide and *Anhydrous sodium carbonate* were purchased from Yongda Chemical Reagent Ltd. Co., Tianjin, China. Hydrochloric acid and Nitric acid were purchased from Jingfeng Chemical Engineering Ltd. Co., Tianjin, China. Ferrous ammonium sulfate, glacial acetic acid, and ammonium acetate were purchased from Haiyi Scientific & Trading Ltd. Co., Shanghai, China. Silver nitrate was purchased from Binhai Chemical Engineering Ltd. Co., Tianjin, China. Sodium thiosulfate was purchased from Ruijing Chemical Reagent Ltd. Co., Tianjin, China. Potassium iodide was purchased from Comeo Chemical Reagent Ltd. Co., Tianjin, China. ZVI was stored in a sealed black airtight bag and placed in an anaerobic box for storage before use. The water solution used for testing is an artificial water distribution system designed to simulate polluted water conditions. It does not contain all the diverse ions present in actual polluted water.

### 2.2. Characterization of ZVI

Scanning electron microscopy (S-4800, Hitachi High-Technologies Corporation, Tokyo, Japan) was used to examine the surface morphology of ZVI surface particles before and after reaction. The test voltage was 5 kV. The samples were dried at 50 °C in vacuum box for 24 h. Specific surface area and distribution of pore size of ZVI were obtained by using specific surface area and pore size analyzer(ASAP-2020, Micromeritics Instrument Corporation, Norcross, GA, USA) The samples were degassed at 30 °C for 4 h, and the N_2_ adsorption volume was measured at 77.249K. Surface area, particle, and pore size were detected with Brunauer–Emmett–Teller (BET) method. Moreover, X-ray diffraction,(XRD-6100 Shimadzu Corporation, Kyoto, Japan) analysis was carried out to assess changes in the crystalline structure of ZVI before and after reaction. (2θ range 10~90°, scan step size of 0.02°, scanning frequency of 8°/min).

### 2.3. Experimental Process

A number of experiments were carried out to compare the degradation and dechlorination efficiency of TCE under various condition. (TCE concentration, dosage of ZVI, H_2_O_2_ concentration, initial pH and temperature). Dissolve a certain volume of TCE standard solution in methanol and then dilute it into 500 mg/L TCE methanol stock solution. Adjust the solution by using 1 mol/L NaOH or HCl. In the present study, solution pH was recorded by a pH meter (PWR-351N, Osho Ltd. Co., Shanghai, China) in all experiments. Vials containing the solution that meets the experimental requirements were transferred to a gas bath thermostatic oscillator, (ED-85A Shanghai Precision Instrument Co., Ltd., Shanghai, China) operating at 150 rpm. TCE in aqueous suspensions was periodically sampled and detected by a Gas Chromatograph–Flame Ionization Detector (Agilent Technologies, Santa Clara, CA, USA), which is equipped with a GS-GasPro (30 m × 0.32 mm) capillary column. The aqueous Cl^−^ concentration in the experimental process was obtained by the silver nitrate titration method. Moreover, a UV spectrophotometerTU-11810, Puxuan General Instrument Ltd. Co., Beijing, China) was used to investigate the aqueous Fe(II) concentration.

The level of hydroxyl radical was measured by fluorescence spectrophotometry to study the mechanisms of ZVI Fenton process. Briefly, 83.0 mg terephthalic acid powder was added into a vial with 2.0 mmol/L sodium hydroxide solution (5.0 mmol/L terephthalic acid). TCE in aqueous suspensions was periodically sampled and filtered with 0.45 μm filter membrane. Fluorescence peak intensity of 2-hydroxyterephthalic acid (Proportional to the level of hydroxyl radical) in the filtrate was detected with fluorescence spectrophotometry.

To ensure accuracy, all experiments were performed in triplicate. The reported data represent the average values from three independent experiments.

## 3. Results and Discussion

### 3.1. Characteristics of ZVI

#### 3.1.1. Surface Morphological Characteristics of SEM

**Figure 1:**
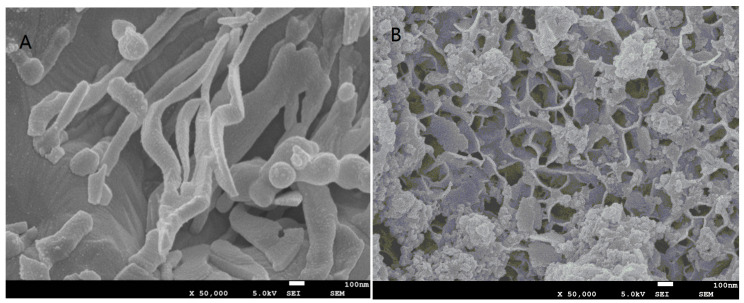
SEM diagrams of zero-valent iron (**A**) before reaction and of zero-valent iron (**B**) after reaction (enlarged 50,000 times).

The SEM image (Figure 1A) shows that ZVI particles are smooth and aggregated into spherical or chain-like structures. After 24 h of reaction, the surface of the ZVI particles becomes rough and porous (Figure 1B), primarily due to surface oxidation, a phenomenon also reported by Peng [31]. The surface of ZVI particles covered with oxidized ZVI was likely to decrease the level of Fe(II), thereby inhibiting TCE degradation. This is attributed to the barrier effect of the oxidized layer, which impedes electron transfer and limits Fe(II) release.

#### 3.1.2. BET Analysis of ZVI

**Figure 2:**
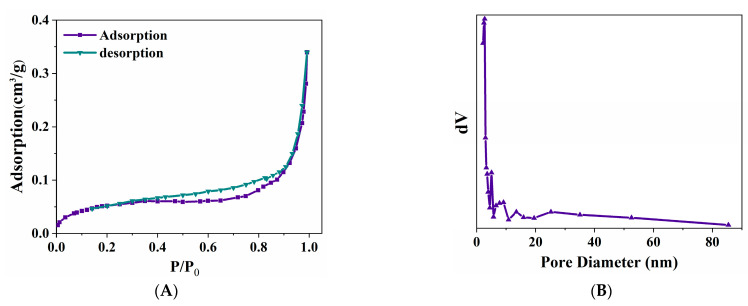
N_2_ adsorption–desorption isotherm of zero-valent iron (**A**) and pore size distribution of zero-valent iron (**B**).

Figure 2A presents the N_2_ adsorption–desorption isotherm of zero-valent iron (ZVI). The N_2_ adsorption–desorption isotherm of ZVI zero-valent iron presents an obvious hysteresis loop. According to IUPAC, it can be determined that the N_2_ adsorption–desorption isotherm of ZVI is type IV, and the hysteresis loop is type H4. This indicates an irregular pore structure with no well-defined adsorption saturation plateau. The BET surface area and pore size distribution were analyzed both before and after the reaction to examine structural changes. Before the reaction, the BET surface area of ZVI was 0.2 m^2^/g, with adsorption and desorption pore sizes of 15.7 nm and 11.7 nm, respectively. The pore size distribution ranged from 2.2 nm to 50 nm, suggesting a mix of mesoporous and non-porous structures (Figure 2B). After the reaction, the BET surface area showed a significant reduction, while the average pore size increased, indicating structural changes due to surface oxidation and pore collapse. This suggests a decrease in available reactive sites, which could affect the reactivity of ZVI.

#### 3.1.3. X-Ray Spectroscopy (XRD) Analysis of ZVI

**Figure 3:**
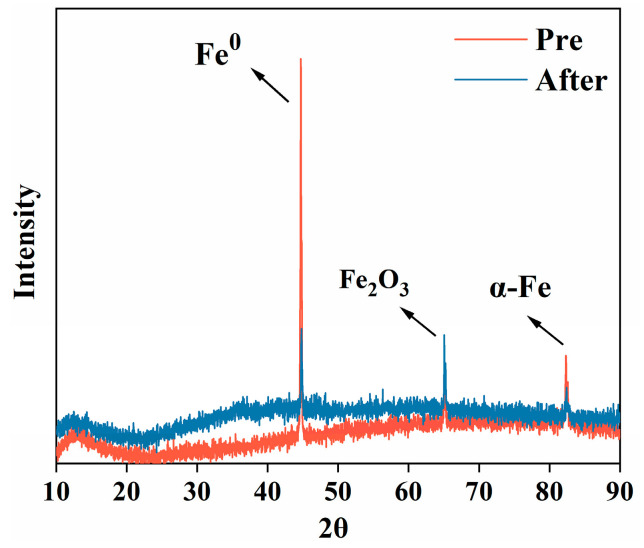
XRD diagram of ZVI before and after Reaction.

The reactivity of ZVI has been reported to be closely related to its surface composition [32]. Therefore, XRD analysis was conducted to characterize ZVI before and after the reaction. The XRD pattern in Figure 3 shows that the peaks of Fe^0^ in the after-reaction ZVI could not be distinctly discerned in the diagram. This is because of its low degree of crystallinity [33]. In contrast, the pre-reaction ZVI exhibits a prominent peak at 45°, indicating a high degree of crystallinity and phase purity (PDF: 06-0696). After the reaction, the X-ray diffraction intensity significantly decreased, which can be attributed to the formation of oxidized ZVI. The peaks corresponding to Fe_2_O_3_ and Fe_3_O_4_ became obvious, and the peak of Fe^0^ receded, which verifies the surface composition and physical and chemical properties transformation of ZVI after reaction. These XRD results are consistent with the surface morphological characteristics observed in the SEM analysis.

### 3.2. Effects of TCE Concentration, Dosage of ZVI, H_2_O_2_ Concentration, Solution Temperature, and Initial pH on the Removal Efficiency of TCE by ZVI/H_2_O_2_

#### 3.2.1. TCE Concentration

**Figure 4:**
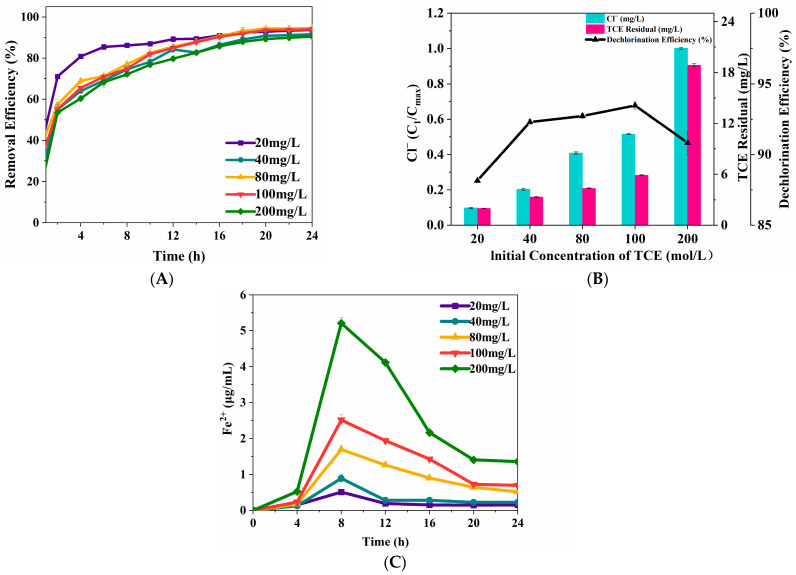
The effects of initial TCE on TCE degradation (**A**) and dechlorination (**B**). Concentration profile of aqueous Fe(II) released from ZVI surface (**C**). (ZVI = 2 g/L, pH = 3, H_2_O_2_ = 0.53 mol/L, T = 25 °C).

TCE degradation and dechlorination ratio as well as the level of aqueous Fe(II) was monitored to evaluate the effect of initial TCE concentration on TCE removal efficiency (including degradation efficiency and dechlorination efficiency) using the ZVI Fenton process. As shown in Figure 4A, the TCE degradation ratio initially increased and then declined with increasing initial TCE concentrations. At an initial TCE concentration of 80 mg/L, the degradation ratio reached its peak at the maximum value of 94.56%. Similarly, the TCE dechlorination trend in Figure 4B aligns with the degradation pattern in Figure 4A, with a maximum dechlorination ratio of 93.47% observed at an initial TCE concentration of 100 mg/L. Figure 4C shows that aqueous Fe(II) concentration was positively correlated with the initial TCE concentration. Aqueous Fe(II) concentration reached its peak at 8 h before gradually decreasing. As the TCE concentration increased 10 times to 200 mg/L, the aqueous Fe(II) concentration increased 9.23 times to 200 mg/L, indicating effect of initial TCE concentration indicating a significant impact on aqueous Fe(II) released from ZVI. However, at 200 mg/L, the TCE degradation and dechlorination ratios increased only by 4.92% and 6.03%, respectively, likely due to surface oxidation of ZVI. At this concentration, ZVI surfaces rapidly became covered with oxidized layers, leading to reaction termination. The reason for the difference in corrosion ratios between oxidized and granular ZVI particles is the formation of passivating precipitates on their surfaces, as previously observed in granular ZVI systems [34].

#### 3.2.2. Dosage of ZVI

**Figure 5:**
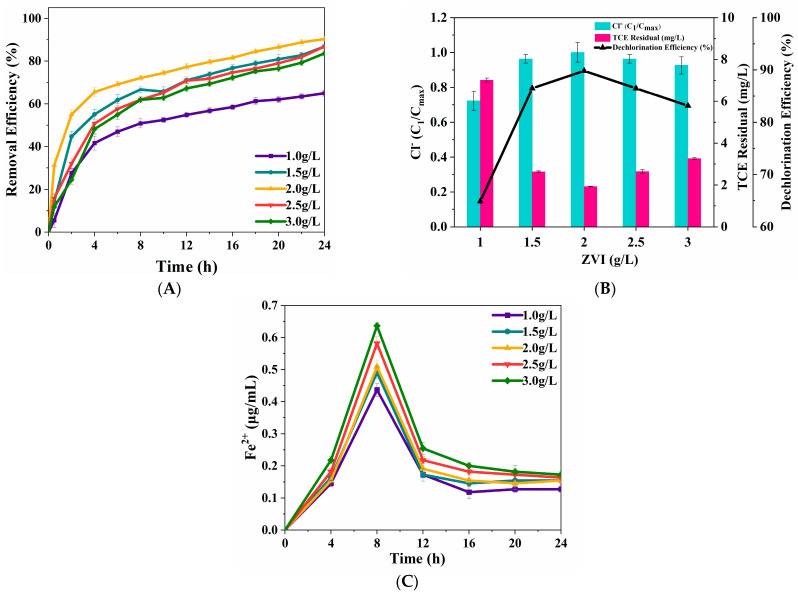
The effects of dosage of ZVI on TCE degradation (**A**) and dechlorination (**B**). Concentration profile of aqueous Fe(II) released from ZVI surface (**C**). (TCE = 20 mg/L, pH = 3, H_2_O_2_ = 0.53 mol/L, T = 25 °C).

Figure 5A shows TCE removal efficiency using Fenton process at five different dosage of ZVI. Initially, the TCE degradation efficiency increased with higher ZVI dosages but declined beyond a certain threshold. After 24 h, the degradation ratio reached 64.97% at 1 g/L ZVI, while at 2 g/L ZVI, it peaked at 90.37%, representing a 39% increase when the ZVI dosage doubled.

Figure 5B indicates that TCE dechlorination ratio increased as the dosage of ZVI increased to 2g/L and reaching the maximum state with an insignificant decrease in degradation ratio. This trend aligns with the findings of Harada et al. [35]. Specifically, when the ZVI dosage doubled to 2 g/L, the dechlorination ratio increased by 38.4%, reaching a maximum value of 89.81%. This can be attributed to the significant presence of aqueous Fe(II) in the system.

Figure 5C demonstrates that the concentration of aqueous Fe(II) consistently increased in correlation with the dosage of ZVI, which indicated that the decrease in degradation ratio (2~3 g/L ZVI) might be caused by a quenching reaction. The level of aqueous Fe(II) continued to increase and then reached a maximum condition as the dosage of ZVI increased to 2g/L. When the active sites of TCE are occupied, the excessive aqueous Fe(II) can consume the ·OH produced during the ZVI Fenton process [36], which is described in Equations (1) and (2). This phenomenon may explain the slight decrease in TCE degradation and dechlorination at higher ZVI concentrations.

2OH + 2OH → 2H_2_O + 2O (1)

H_2_O_2_ + ·OH → HO_2_· + H_2_O (2)

#### 3.2.3. H_2_O_2_ Concentration

**Figure 6:**
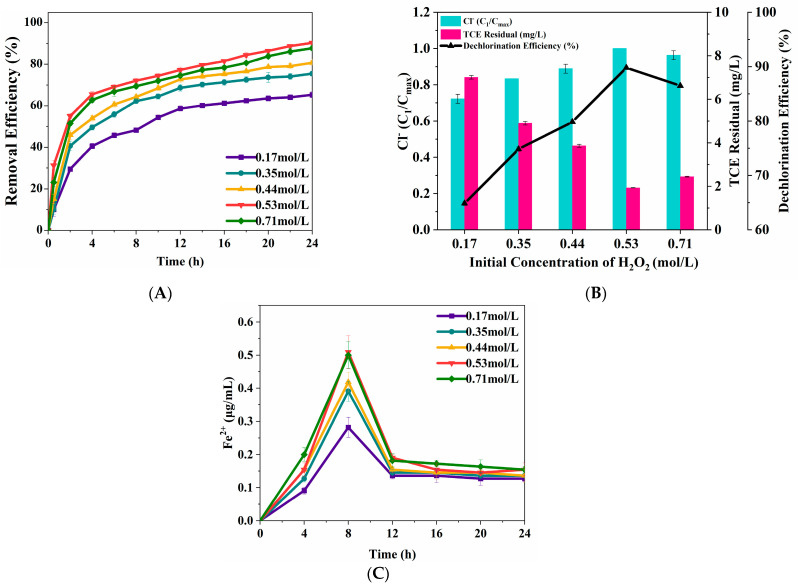
The effects of hydrogen peroxide concentration on TCE degradation (**A**) and dechlorination (**B**). Concentration profile of aqueous Fe(II) released from ZVI surface (**C**). (TCE = 20 mg/L, ZVI = 2g/L, pH = 3, T = 25 °C).

Figure 6A indicates oxidative degradation of TCE with time under different concentrations of hydrogen peroxide. The TCE degradation efficiency initially increased with rising H_2_O_2_ concentrations but declined beyond a certain threshold. TCE degradation ratio reached 65.34% at 0.17 mol/L H_2_O_2_, while TCE degradation increased by 38.31% and reached the maximum value of 90.37% at 0.53 mol/L H_2_O_2_. TCE dechlorination diagram showed in Figure 6B showed an increased trend initially until the concentration of H_2_O_2_ increased to 0.53 mol/L. When the hydrogen peroxide concentrations were 0.17 mol/L and 0.53 mol/L, respectively, the dechlorination ratios of TCE were 69.86% and 89.81%, respectively. The latter increased by 38.46% compared with the former. The level of aqueous Fe(II) increased by 81.13% (0.509 mol/L) as the concentration of H_2_O_2_ increased from 0.17 mol/L to 0.53 mol/L (Figure 6C), indicating that increasing the concentration of H_2_O_2_ could significantly increase aqueous Fe(II) released from the ZVI surface, thereby expanding the reaction area of H_2_O_2_. Additionally, an increase in the concentration of H_2_O_2_ could result in a higher abundance of hydroxyl radicals as a source. The concentration of hydroxyl radicals increases, thereby improving the efficiency of the reaction. However, the removal of TCE showed a decrease in the removal trend at a higher concentration of H_2_O_2_, which might be due to the excessive hydroxyl radicals released from H_2_O_2_. Once TCE active sites become saturated, excessive hydroxyl radicals could react with H_2_O_2_ and form hydroperoxide radicals [37]. These hydroperoxide radicals can subsequently react with hydroxyl radicals, leading to the consumption of hydroxyl radicals. The reaction between hydroperoxide radicals and hydroxy radicals is described in Equation (3).

HO_2_·+·OH—H_2_O + O_2_ (3)

#### 3.2.4. Solution Temperature

**Figure 7:**
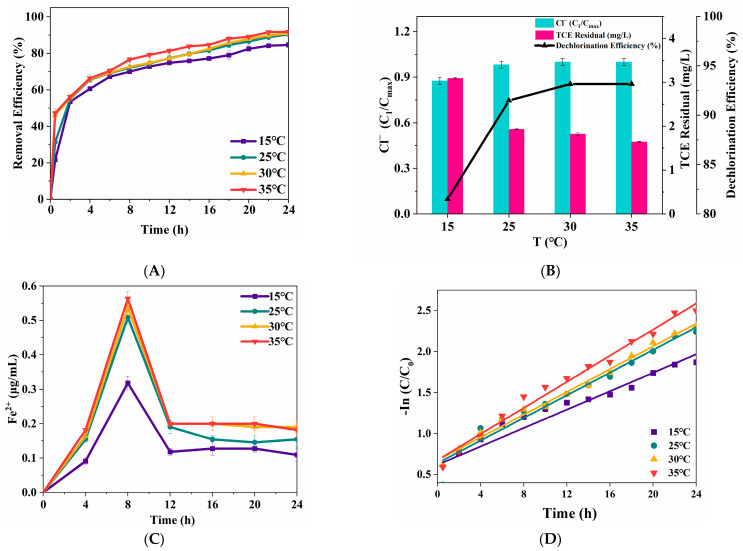
Effects of temperature on TCE degradation (**A**) and dechlorination (**B**). Concentration profile of aqueous Fe(II) released from the ZVI surface (**C**). Variation in kinetic constants of different temperature contents (**D**) (TCE = 20 mg/L, ZVI = 2 g/L, pH = 3, H_2_O_2_ = 0.53 mol/L).

Temperature plays a critical role in the Fenton oxidation reaction, influencing the reactivity of ZVI toward contaminants. The removal efficiency of TCE was detected to study the efficiency of solution temperature on the degradation ratio of TCE, and the results were showed in Figure 7A. Using the ZVI Fenton process, the degradation ratio was greatly inhibited in the first hour under solution temperature of 15 °C and 25 °C. However, by the end of the reaction, the degradation ratio only increased by 6.8% when the temperature rose from 15 °C to 25 °C, and no further significant increase was observed at higher temperatures. It was found that the TCE removal efficiency seemed to be slightly influenced by solution temperature. TCE dechlorination efficiency was examined to further investigate the influence of solution temperature on TCE removal efficiency by ZVI/H_2_O_2_. As shown in Figure 7B, the TCE dechlorination ratio varyingly increased as solution temperature increased from 15 °C to 25 °C, while, the dechlorination ratio reached a limiting value with solution temperature of 30 °C and kept constant from 30 °C to 35 °C. Notably, these trends contrast with the findings of Metzgen [38]. It seemed that the degradation efficiency and dechlorination efficiency could not represent the TCE removal efficiency in the ZVI Fenton process.

To better understand the reaction mechanism, the kinetic parameters of TCE degradation were analyzed (Figure 7D, Table 1). The kinetic parameters of TCE removal by ZVI/H_2_O_2_ showed an increased trend as the solution temperature increased, indicating that solution temperature was in direct proportion to TCE removal efficiency, and the removal experiment was an endothermic reaction, in which kinetic energy of molecules increased as the solution temperature increased, intensifying the collision between H_2_O_2_ and aqueous Fe(II). Similar result was also observed by Fan [39]. However, considering the process cost, the solution temperature of 25 °C is enough to maintain a high reactivity of ZVI/H_2_O_2_ during the process.

#### 3.2.5. Initial pH

**Figure 8:**
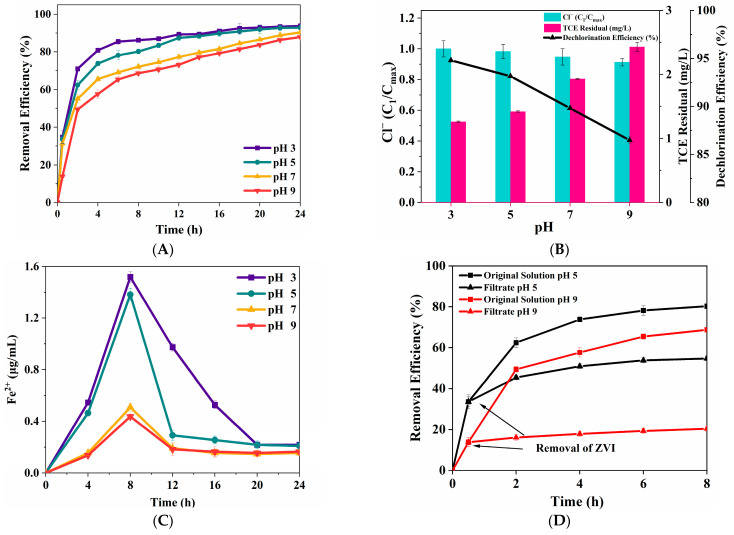
The effects of initial pH on TCE degradation (**A**) and dechlorination (**B**), Concentration profile of aqueous Fe(II) released from ZVI surface (**C**), Heterogeneity tests (**D**) (TCE = 20 mg/L, ZVI = 2 g/L, H_2_O_2_ = 0.53 mol/L, T = 25 °C).

Previous studies have demonstrated that TCE removal efficiency by the ZVI Fenton process varies by different experimental substrates [28,29,30]. Therefore, it is of great value to study the degradation efficiency of TCE influenced by initial pH (Figure 8). The results indicate that TCE removal was only slightly affected by initial pH, as all tested conditions resulted in over 90% removal after 24 h. The trend of TCE removal with pH was consistent with a previous study [29]. TCE degradation slightly decreased as the solution pH increased to pH 3, 5, 7, and 9. The degradation ratio reached the maximum value of 93.71% at pH 3. To further examine the effect of solution pH on TCE removal, TCE dechlorination was monitored. Figure 8B shows that TCE residual increased as the solution pH increased, which is consistent with TCE degradation. The dechlorination ratio reached the maximum value of 94.80% at pH 3, and the minimum value of 86.48% at pH 9. Figure 8C illustrates that the level of Fe(II) decreased as the solution pH increased, the level of Fe(II) reached the maximum concentration of 1.518 μg/mL far beyond pH of 5, 7, 9. The reactivity of ZVI is highly sensitive to solution pH, primarily due to the surface of ZVI covered with oxidized ZVI (Fe_2_O_3_, Fe_3_O_4_ [31]) under alkaline conditions. Conversely, under acidic conditions, ZVI undergoes rapid corrosion, leading to higher reactivity [40]. A similar pattern was also observed in the study carried out by Choi, in which explained that the TCE removal ratio by ZVI Fenton with Cu(II) decreased with solution pH from 3 to 6, which might be caused by a decrease in the reduction potential of reactive surface species as the increase in solution pH occurs [26]. Therefore, under acidic conditions, ZVI can supply sufficient Fe(II) to catalyze the decomposition of H_2_O_2_ into active free radicals, promoting the removal of TCE. Under alkaline conditions, H_2_O_2_ tends to behave as a reductant, which may reduce its oxidative capacity and consequently lower the removal efficiency. Moreover, under alkaline conditions, the level of hydroxyl radicals will reduce because OH^−^ can consume H_2_O_2_, leading to a low TCE dechlorination ratio.

Heterogeneity tests were applied to discriminate between homogeneous (aqueous Fe^2^⁺) and heterogeneous (ZVI surface-mediated) pathways, specifically assessing whether Fe^2^⁺ remains active after physical separation from ZVI. ZVI was removed from the reaction mixture by a pre-warmed 0.1 μm PTFE membrane after a reaction time of 0.5 h, and filtration occurs for another 7.5 h under identical conditions. As shown in Figure 8D, the removal efficiencies of TCE in the filtrate under pH 5 and pH 9 conditions were 54.27% and 20.42%, respectively. The TCE in the filtrate of pH 5 continued to degrade, which corresponded to 68% of the removal efficiency observed in the original solution, indicating that the catalytic reaction is a homogeneous process under acidic conditions. Under alkaline conditions (pH 9), minimal TCE degradation was observed in the filtrate, which corresponded to 29% of original solution.

### 3.3. TCE Degradation Mechanisms in ZVI Fenton Process

**Figure 9:**
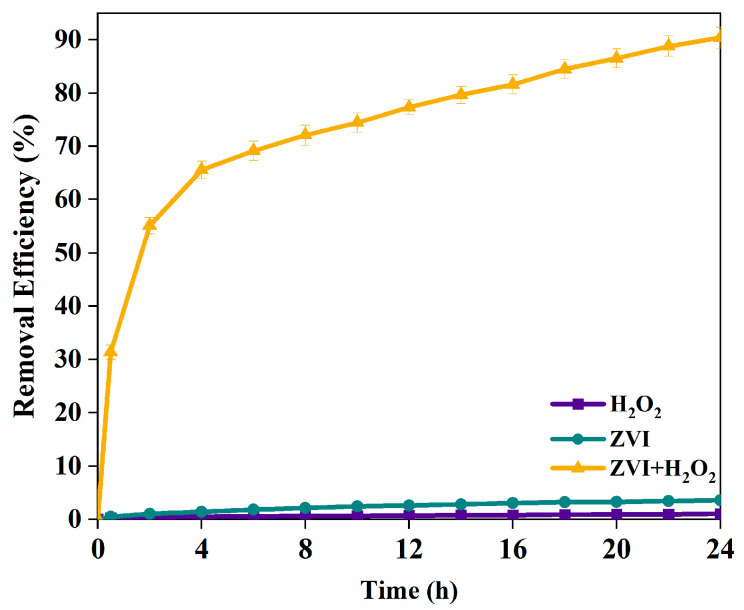
Effect of different addition materials on TCE degradation ratio. (TCE = 20 mg/L, ZVI = 2 g/L, H_2_O_2_ = 0.53 mol/L, pH = 7, T = 25 °C).

Figure 9 indicates the variation in TCE removal efficiency at different addition materials reactions. Reaction with bare H_2_O_2_ did not show any significant loss of TCE after 24 h, suggesting no significant Fenton oxidation throughout the reaction. This is because hydrogen radical generation from H_2_O_2_ alone, without a catalyst, was insufficient to effectively degrade TCE. There was only a slight decrease in TCE by bare ZVI, and the removal of TCE was mainly attributed to minor adsorption. A similar pattern was also observed by H. Dong [30]. The removal of TCE efficiency by ZVI/H_2_O_2_ reached 91.37%, verifying that TCE could be degraded efficiently in the ZVI Fenton process. In order to examine the production of hydroxyl radical, fluorescence spectroscopy detections using terephthalic acid as probes were carried out. The results are included in the Supplementary Materials.

## 4. Conclusions

This study examined the effects of TCE concentration, ZVI dosage, H_2_O_2_ concentration, initial pH, and temperature on the removal efficiency of TCE in the ZVI-Fenton process. Additionally, TCE degradation mechanisms and ZVI characterizations before and after reactions were analyzed. The findings indicate that while TCE concentration had a limited effect on degradation efficiency, ZVI dosage and H_2_O_2_ concentration significantly influenced removal ratios. The process demonstrated high efficiency (>90%) across a wide pH range (3–9), suggesting its potential applicability for groundwater purification. Reaction temperature positively correlated with TCE degradation ratios. SEM analysis reveal that the surface of ZVI particles after the reaction became rough and porous and had consisted of surface-oxidized ZVI, which is also observed by Zhao et al. [41]. XRD analysis shows that the surface crystallinity of ZVI is low, which could decrease the enhanced reactivity. Future research should extend these findings by conducting experiments using real water samples rather than aqueous solutions, as natural water sources contain various compounds that may compete with TCE for degradation, which is also supported by Guo et al. [42]. Investigating the influence of environmental factors such as pH, temperature, and organic matter on degradation efficiency will further enhance practical applications.

## Figures and Tables

**Table 1:** Kinetic parameters of TCE degradation.

T (°C)	Reaction Kinetics Constant (/h)	R^2^
15	0.0947	0.9760
25	0.1250	0.9902
30	0.1347	0.9907
35	0.1537	0.9846

## Data Availability

Data will be made available on request.

## Supplementary Materials

The following supporting information can be downloaded at: https://www.mdpi.com/article/10.3390/nano15070558/s1, Figure S1: Fluorescence spectra of terephthalic acid in ZVI Fenton system. (TCE = 20 mg/L, ZVI = 2 g/L, H_2_O_2_ = 0.53 mol/L, pH = 7, T = 25 °C).
