# Supplementary material for: Factors Influencing Removal of Trichloroethylene in a Zero-Valent Iron Fenton System
Source: Nanomaterials (Basel). 2025 Apr 5;15(7):558. doi: 10.3390/nano15070558 (PMC11990697; doi:10.3390/nano15070558)
Supplement: Supplementary file 1 [file nanomaterials-15-00558-s001.zip › nanomaterials-3525915-supplementary.pdf]

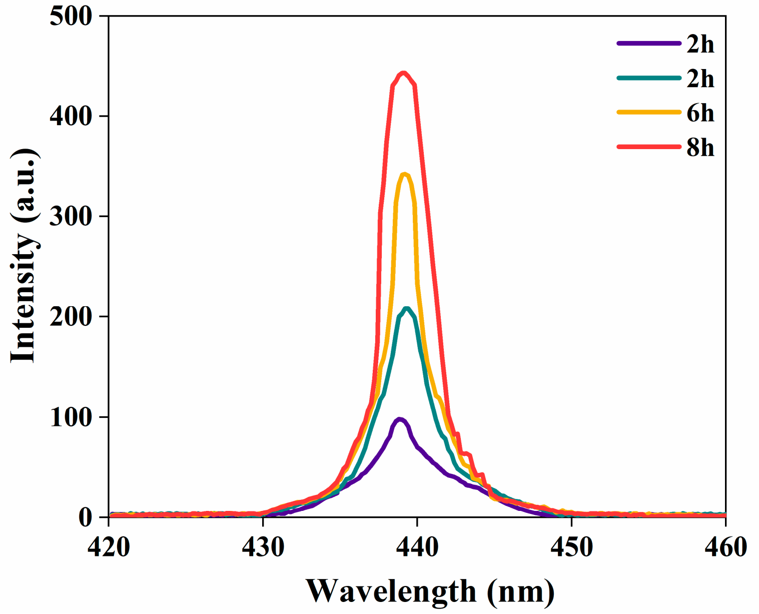

Figure S1. Fluorescence spectra of terephthalic acid in ZVI Fenton system. (TCE=20mg/L, ZVI=2g/L, H_2O_2=0.53mol/L, pH=7, T=25℃)

In order to examine the production of hydroxyl radical, fluorescence spectroscopy detections using terephthalic acid as probes were carried out and shown in Figure S1. The maximun fluorescence intensity was detected at 439nm, indicating the presence of hydroxyl radicals in the reaction. The maximun fluorescence increased by 3.5 times from 97.76 to 443.58 as the reaction time increased (8h). This is because ZVI acted as a slow release source of aqueous Fe (II), thereby catalyzing the release of hydroxyl radical radicals.
